# Percutaneous ethanolamine oleate sclerotherapy for aggressive vertebral hemangioma: A case report

**DOI:** 10.1016/j.radcr.2023.06.017

**Published:** 2023-06-20

**Authors:** Masayuki Endo, Shuichi Yamamoto, Shinsaku Yata, Shohei Takasugi, Kazumichi Tsukamoto, Jun Makishima, Yuji Kamata, Misato Kishimoto, Kentaro Shinano, Shinya Fujii, Yasufumi Ohuchi, Shinji Tanishima

**Affiliations:** a Division of Radiology, Department of Multidisciplinary Internal Medicine, Tottori University, 36-1 Nishi-cho, Yonago Tottori 683-0854 Japan; b Department of Radiology, Matsue Red Cross Hospital, 200 Horomachi, Matsue, Shimane, Japan; c Division of Orthopedic Surgery, Department of Sensory of Motor Organs, School of Medicine, Faculty of Medicine, Tottori University, Yonago Tottori, Japan

**Keywords:** Vertebral hemangiomas, Percutaneous ethanolamine oleate, Sclerotherapy

## Abstract

Vertebral hemangiomas are the most common benign lesion of the spine which are often an asymptomatic incidental finding. However, a few hemangiomas are aggressive and characterized by bone expansion and extraosseous extension into the paraspinal and epidural spaces. We report the case of a patient presenting an aggressive vertebral hemangioma causing back pain and bilateral numbness of the legs. Among various treatment modalities, a minimally invasive percutaneous sclerotherapy procedure using ethanolamine oleate under computed tomography and fluoroscopic guidance was safely and successfully performed with good clinical outcomes.

## Introduction

Vertebral hemangiomas are benign vascular lesions of the spine with a prevalence of 10%-12% in the general population, and accounts for approximately 2%-3% of all spinal tumors [1] and are mostly asymptomatic incidental findings. However, a few hemangiomas are aggressive, characterized by bone expansion and extraosseous extension into the paraspinal and epidural spaces [2] causing pain and neural compression due to vertebral fracture and compression of nerve root or dural sac, respectively [1,3].

Surgical therapy has been the treatment of choice for aggressive vertebral hemangiomas for many years. However, this therapy has been associated with major perioperative blood loss. Recently, various treatment options have been suggested including endovascular or percutaneous embolization, percutaneous vertebroplasty, and radiotherapy as an alternative to the traditional surgical treatment.

We report the case of a 79-year-old patient presenting with symptomatic aggressive vertebral hemangioma of the lumber spine, who was successfully treated with a minimally invasive percutaneous ethanolamine oleate sclerotherapy under computed tomography (CT) and fluoroscopic guidance.

## Case report

**Fig. 1:**
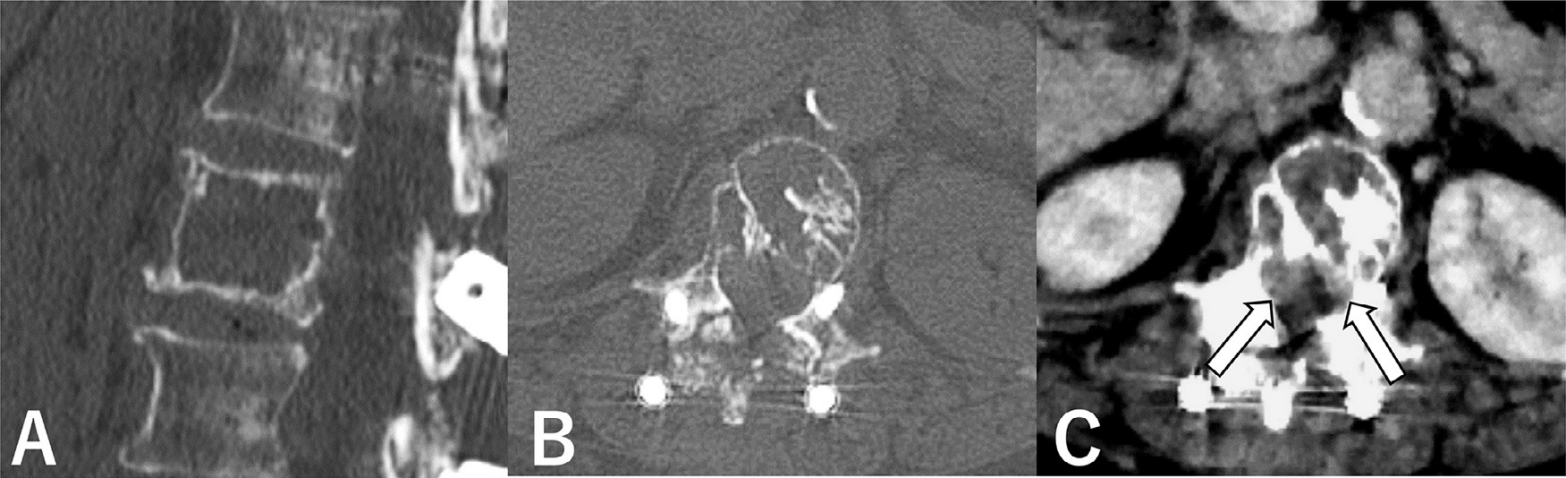
Aggressive vertebral hemangioma involving the L1 vertebral body in a 79-year-old female with lower back pain and symptoms of neurological compression. (A, B) Sagittal and axial CT showing an expansive and osteolytic lesion in the entire vertebral body of L1 with erosion of the posterior vertebral wall. (C) Axial contrast-enhanced CT showing an enhancing lesion involving the entire vertebral body. The lesion extends into the anterior epidural space (arrows), causing compression of the cauda equina. CT, computed tomography.

A 79-year-old female presented to the hospital with back pain and bilateral lateral thigh numbness that gradually worsened over 8 years. Computed tomography showed an expansile and osteolytic lesion (33.7 × 33.9 × 20.0 mm), in the L1 vertebral body. The lesion extended into the anterior epidural space, causing compression of the cauda equina (Fig. 1). The lesion was initially discovered on CT 7 years previously and was considered to be a slow-growing tumor. Based on the clinical course and imaging findings, an aggressive vertebral hemangioma with an epidural component was suspected. Magnetic resonance imaging (MRI) was not performed as the patient had a pacemaker. Posterior fixation was previously performed to achieve decompression and prevent compression fracture, however, the patient's symptoms did not improve. The patient was then referred to our department for treatment using interventional techniques, and percutaneous sclerotherapy was planned.

After consultation with multidisciplinary team, percutaneous sclerotherapy was performed. This procedure was approved by our institutional review board, and the patient provided written informed consent.

**Fig. 2:**
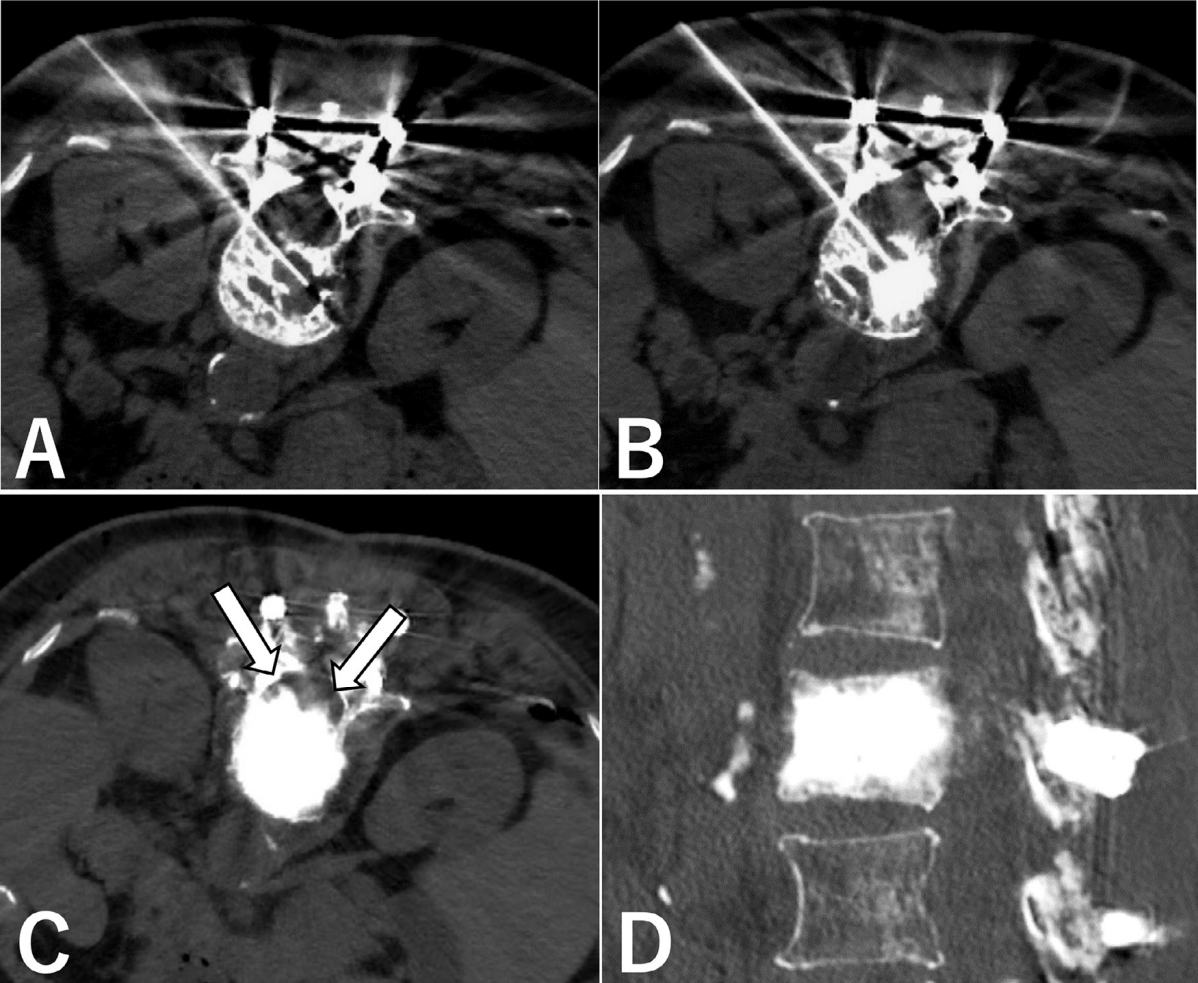
Fluoroscopy and CT-guided intraosseous sclerotherapy. (A) A puncture needle was introduced within the vertebral body by a posterolateral approach. (B) Five percent ethanolamine oleate mixed with contrast media was injected into the lesion. (C, D) Sclerosant is well distributed to the epidural component (arrows) and in the entire vertebral lesion on postoperative CT image. CT, computed tomography.

The procedure was performed in the prone position under local anesthesia (10 mL of 1% lidocaine) in an angiographic suite equipped with a hybrid angio-CT system (Aquilion PRIME, TSX-303A/BW; Canon Medical Systems, Tochigi, Japan). Under CT guidance, a direct posterolateral approach with a 19-gauge coaxial introducer needle (ARGON medical device, TX) was followed. The inner needle was withdrawn while holding the cannula in place after confirming correct needle placement using CT. Following this, 2 mL of liquid sclerosant (5% ethanolamine oleate with contrast media) was injected slowly and carefully into the lesion under fluoroscopy. CT was performed immediately after the injection to ensure intralesional spread of the opacified sclerosant. The cannula was repositioned in different areas to allow homogenous distribution, and 2 mL of sclerosant was injected in each position. A total of 8 mL of 5% ethanolamine oleate was injected, and the final CT revealed an optimal distribution within the vertebral body and epidural space without leakage (Fig. 2). Finally, gelatin sponge particles were then injected through the cannula to prevent reflux of the sclerosant, and the cannula was removed.

**Fig. 3:**
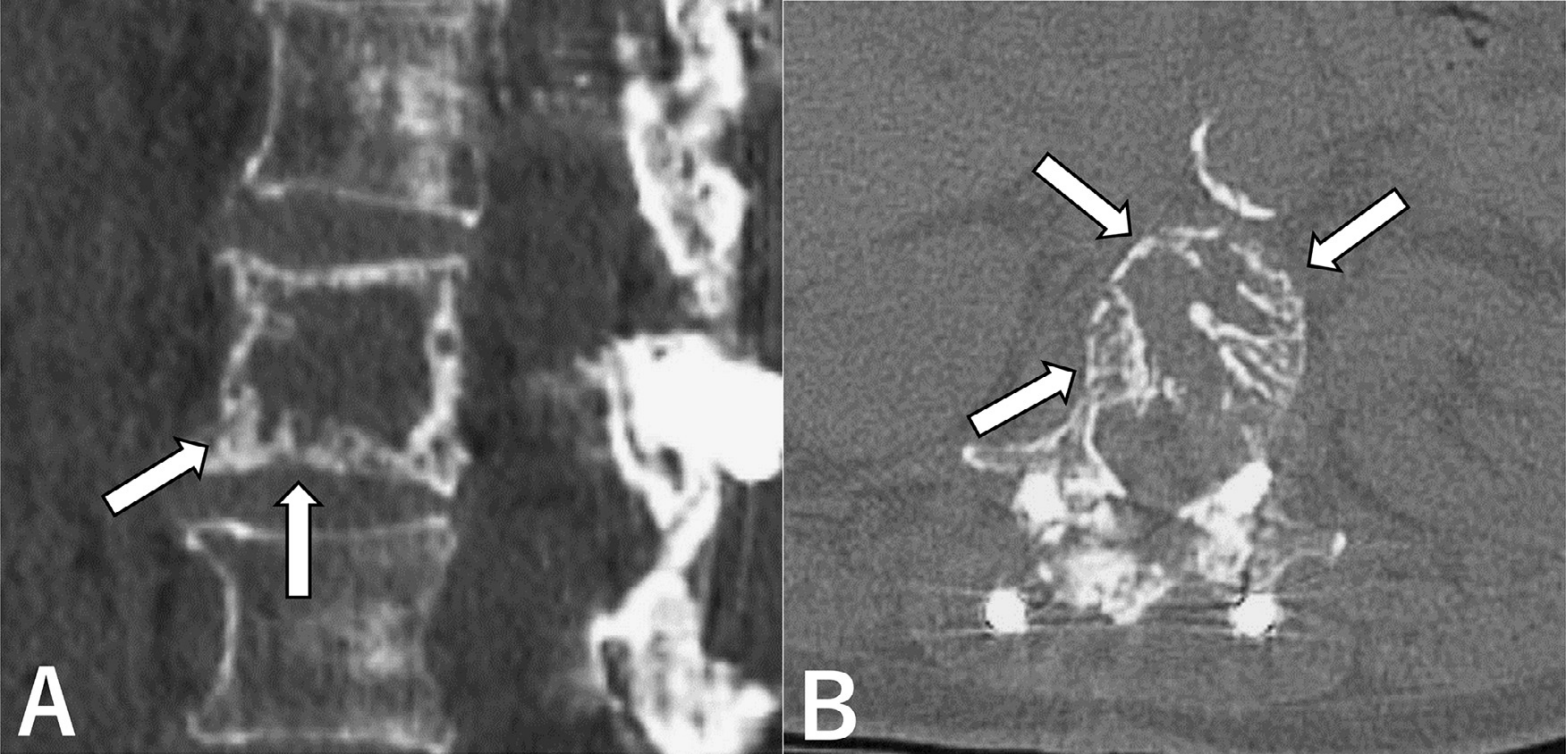
One-year follow-up CT scans. (A, B) The texture of the vertebral centrum partially regains its normal appearance (arrows). CT, computed tomography.

The patient experienced mild back pain and slight fever after the procedure and received potent analgesics to resolve the pain. The patient was discharged 2 days after the procedure. At 1-year follow-up, the patient's back pain was greatly relieved and bilateral lateral thigh numbness had improved and returned to normal. Follow-up CT demonstrated the L1 vertebral body had partially regained its normal texture and appeared denser, reflecting osteogenesis (Fig. 3).

## Discussion

Vertebral hemangiomas can be either cavernous or capillary, with the former being more common in the body of the vertebra and characterized by venous engorgement with large sinusoidal spaces [4]. Generally, vertebral hemangiomas are incidental findings in asymptomatic patients. Symptomatic vertebral hemangiomas account for 0.9%-1.2% of cases in the general population, and the clinical presentation may vary from pain to radicular symptoms and myelopathy [4,5]. Pain is often due to fracture of the involved vertebral body or canal stenosis caused by bone expansion, whereas myelopathy is observed in association with epidural extension of the lesion.

CT can provide detailed findings of the microstructure of the lesion, including the typical “polka-dot” sign, and nonspecific findings, such as ballooning or lysis of the cortex [6] or extraosseous soft tissue extension [7,8]. In the present case, the typical “polka-dot” sign, which can aid in correct diagnosis, was not clearly observed on CT because of the destructive nature of the lesion. Additionally, our patient did not undergo MRI due to the use of a pacemaker and was not diagnosed histologically. However, the CT findings and clinical features such as slow progression and good prognosis after the treatment procedure were compatible with a diagnosis of aggressive vertebral hemangioma.

Numerous treatment options and various combinations of these have been described for symptomatic aggressive vertebral hemangiomas. Although urgent decompressive surgery is widely accepted as the treatment of choice for acute spinal cord compression or cauda equina syndrome, the best treatment option for aggressive vertebral hemangiomas with progressive neurological impairment remains controversial.

Percutaneous sclerotherapy for aggressive vertebral hemangiomas has several advantages. First, it is less invasive than surgical resection. Second, prolonged immobilization of the patient is not necessary. Third, it can be repeated, if necessary, to eradicate the residual hemangioma. Regarding the sclerosant, we used 5% ethanolamine oleate because it is effective in vascular lesions resembling hemangiomas, such as varices, and is superior to polidocanol for intravascular injection [9]. In contrast to absolute ethanol, which clears rapidly after injection, the relatively slow diffusion of the opacified sclerosant within the lesion can be easily followed during the procedure. Additionally, ethanol injections have a risk of causing neurological complications. Niemeyer et al. [10] described a case of Brown-Sequard syndrome following ethanol injection, which may have been due to a retrograde leak that was undetected during the percutaneous injection. In the present case, the combination of fluoroscopic and CT guidance during the injection allowed strict monitoring of the spread of the opacified sclerosant within the vertebral body and extraosseous component, which prevented migration into undesirable regions.

In conclusion, percutaneous ethanolamine sclerotherapy is a minimally invasive safe, and effective therapeutic option for treating symptomatic aggressive vertebral hemangioma with epidural involvement.

## Ethical approval

For this type of study formal consent is not required.

## Consent for publication

Consent for publication was obtained for every individual person's data included in this study.

## Patient consent

Written informed consent was obtained from the patient prior to submission of this case report.
